# Genetic testing in the diagnosis of cerebral palsy: First‐tier for all versus a ‘choosing wisely’ approach

**DOI:** 10.1111/dmcn.16327

**Published:** 2025-04-19

**Authors:** Ingeborg Krägeloh‐Mann

**Affiliations:** ^1^ Department of Developmental and Child Neurology, Social Pediatrics University Children's Hospital Tübingen Germany

## Abstract

This commentary is on the original article by Tantsis et al. on pages 1443–1452 of this issue.

Cerebral palsy (CP) is an umbrella term for a group of non‐progressive disorders involving movement, posture, and motor function. Its definition/diagnosis is based on phenomenology (clinical picture and history), not on aetiology.^1^ With advances in genetic diagnostics, the genetic contribution to CP is increasingly discussed and the question arises about the role of genetic testing in the diagnosis of CP. Recommendations are inconsistent: to opt for a ‘choosing wisely’ strategy on the basis of phenotypical markers or do whole exome sequencing as a first‐tier test in all children with CP.^2^

Tantsis et al. provide data to support the ‘choosing wisely’ approach.^3^ In their study, they started with looking at current practice in a survey of clinicians, and collected expert opinion on which clinical and investigation factors were predictive of a monogenic cause of CP. They then used the same clinical and investigation factors in a retrospective cohort of 100 children with CP, comparing those with a confirmed genetic aetiology to those considered to be non‐genetic. With this mixed methodology, they found that dysmorphic features, intellectual disability, and ‘magnetic resonance imaging (MRI) not compatible with the clinical picture’ are most supportive of a genetic cause of CP. Thus, these phenotypical markers can help to decide who should be tested.

The paper has some drawbacks, which the authors acknowledge. Their cohort was not population‐based and biased by a tertiary care center selection (as are most studies on genetic testing in CP). Children born at term, for example, were over‐represented. Patients with regression (e.g. loss of acquired skills) were not excluded if they also met criteria for CP (while CP definitions usually exclude progressive disorders). And hypotonic CP, which they included, is not accepted as a CP subgroup by many medical communities. However, this does not detract from the validity of their main conclusion, that phenotypical markers including MRI findings are important for deciding whom to test.

A strong point of the study is the systematic inclusion of neuroimaging findings, as this has rarely been reported so far in genetic studies on CP. It is noteworthy that the authors state normal imaging alone is not adequate for decision‐making. Rather, they think that an ‘MRI not compatible with the clinical picture’ should also guide decision‐making. Which means an MRI finding not reflecting the severity of motor impairment in combination with the comorbidities of the child should lead to genetic testing. Or, conversely, it should not lead to testing if an injury/damage in its topography and extent does explain the clinical picture. In fact, when considering population‐based data on MRI in children with CP, cerebral injuries accounting for the clinical picture are present in around 70% of cases.^4^ And the decreasing CP prevalence, shown for Europe and Australia, also argues for a considerable part of CP being acquired.^5^ This underlines that their ‘choosing wisely’ recommendations on the basis of phenotypical markers including neuroimaging have clear implications for clinical practice. For example, not to test children with a very low probability for a genetic cause whose MRI show a clear lesional pattern explaining their disability (unless there is a family history and/or causative external factors are missing). Otherwise, a large proportion of children with CP would get testing with negative results, which clearly is not cost‐effective. It would be very interesting to pursue this issue further in prospective studies, as the authors suggest. There is the possibility of collaboration with Australian population‐based CP registers, which collect phenotypical data on children with CP including neuroimaging.

## Data Availability

Not required.
